# Effects of the Molding Method and Blank Size of Green Body on the Sintering Densification of Magnesia

**DOI:** 10.3390/ma12040647

**Published:** 2019-02-21

**Authors:** Endong Jin, Jingkun Yu, Tianpeng Wen, Chen Tian, Zhaoyang Liu, Beiyue Ma, Feixiong Mao, Lei Yuan

**Affiliations:** 1 School of Metallurgy, Northeastern University, Shenyang 110819, China; jinendong1989@163.com (E.J.); yujk@smm.neu.edu.cn (J.Y.); wentianpeng@outlook.com (T.W.); tianchen199301@163.com (C.T.); 1510242@stu.neu.edu.cn (Z.L.); maby@smm.neu.edu.cn (B.M.); 2 Key Laboratory of Marine Materials and Related Technologies, Zhejiang Key Laboratory of Marine Materials and Protective Technologies, Ningbo Institute of Materials Technology and Engineering, Chinese Academy of Sciences, Ningbo 315201, China; maofx@mail.neu.edu.cn

**Keywords:** magnesia, blank size, vacuum compaction, conventional compaction, entrapped air

## Abstract

The bulk density of sintered magnesia is significantly influenced by molding methodology and blank size of the green body during dry pressing. The entrapped air in the green body plays a critical role in determining the bulk density of magnesia samples. Herein, high-density magnesia samples, with different sizes, are prepared by using vacuum compaction molding and conventional compaction molding. The physical properties, such as bulk density and pore size distribution, and morphology or as-sintered magnesia samples were characterized by using Archimedes method, mercury porosimetry, and scanning electron microscopy (SEM). The results indicate that the bulk density of conventional compaction magnesia samples decreased below 3.40 g·cm^−3^ with the increase of thickness due to the presence of entrapped-air induced large pores and intergranular cracks. In addition, the large pores and intergranular cracks in conventionally-compacted samples are observed by SEM images. However, vacuum compaction of magnesia samples resulted in a bulk density of higher than 3.40 g·cm^−3^ for all thicknesses. Moreover, the defects in vacuum-compacted magnesia samples are mainly in the form of small circular pores.

## 1. Introduction

The sintered magnesia is an important basic refractory raw material and its products are widely utilized in metallurgy and chemical industry due to its excellent properties, such as high melting point and strong slag corrosion resistance [1,2]. It has been reported that the quality of magnesia products is significantly influenced by bulk density of the sintered magnesia. However, the phaneric texture of magnesia hinders the densification of magnesia during sintering process [3]. Recently, several studies have been carried out to increase the bulk density of sintered magnesia. In particular, researchers have aimed to attain a bulk density of more than 3.40 g·cm^−3^, which is considered as a benchmark for high-quality magnesia.

The conventional magnesia sintering processes, which are carried out at 1800 °C, yield the bulk density of 3.29–3.34 g·cm^−3^. Hence, different approaches have been adopted to enhance the bulk density of magnesia, such as higher sintering temperature and utilization of sintering aids [4]. However, high-temperature sintering is not a financially viable approach from the industry’s viewpoint. On the other hand, the addition of sintering aids, such as TiO_2_, Y_2_O_3_, and WO_3_, can reduce the sintering temperature and enhance the bulk density of magnesia [5,6,7,8]. However, the uniform mixing of active material and sintering aid is a challenging task and, in addition, sintering additives can react with magnesia and form a large amount of liquid phase, which reduces the slag erosion resistance of magnesia. Actually, the densification of magnesia is hindered by the physical and chemical properties of the starting raw materials. For instance, the light-burned magnesia, which is usually used to synthesize sintered magnesia, is obtained by calcining magnesite. However, porous microcrystalline aggregates, referred as pseudo phase particles, are easily formed during the magnesite calcination, which hinders the densification of magnesia during sintering process [9,10]. In order to break the pseudocrystalline structure and obtain high-density sintered magnesia, a highly active magnesia powder has been synthesized by using light calcination of hydration of magnesite, which resulted in a high bulk density (3.40 g·cm^−3^) of sintered magnesia [11].

However, the bulk density of 3.40 g·cm^−3^ cannot be attained at industrial scale due to the large size of molded green body and thickness of as-prepared magnesia pellets (8–12 mm). In general, the blank density of large-sized dry-molded green bodies is not uniform due to air entrapment between fine powders. The particles move towards each other under high pressure during sintering, but part of the entrapped air cannot flow out. As a result, a higher pressure is experienced inside the pores, which leads to the formation of internal defects, such as macro-pores and microcracks [12,13]. These defects cannot be eliminated by high-temperature sintering and adversely influence the bulk density of magnesia.

Herein, the bulk density of different-sized sintered magnesia has been studied under two molding techniques and mechanistic insights into the influence of entrapped air on which properties of the sintered magnesia have been provided. The influence mechanism of the entrapped air on the properties of the sintered magnesia was discussed.

## 2. Experimental

### 2.1. Raw Material

The raw material used in this study was the high-activity magnesia powder, which prepared by the following process. The magnesite was calcined at 850 °C for 1 h to obtain the light burned magnesia. Then, the as-received powder was mixed with water and ball-milled for 12 h to obtain magnesium hydroxide. Finally, the magnesium hydroxide was calcined at 850 °C for 3 h to obtain the high-activity magnesia. The chemical composition of magnesite is presented in Table 1.

### 2.2. Experimental Process

The as-prepared active magnesia powders were uniaxially pressed by using the dry pressing technology. Different amounts of active magnesia powder were loaded into the die and green bodies, with a diameter of 20 mm, were obtained by vacuum compaction and conventional compaction. In vacuum compaction, the diffused air was pumped out from the magnesia powder during the formation process and the samples were dry pressed under a pressure of 300 MPa for 60 s. Then, the thickness of the as-prepared green bodies was measured, followed by sintering at 1600 °C for 2 h in air. In conventional compaction, the diffused air was not pumped out and it remained inside the magnesia powder during formation process.

### 2.3. Characterization and Analysis

The morphology of the as-sintered magnesia samples was observed by field emission scanning electron microscopy (FESEM, SU8010N, HITACHI, Tokyo, Japan). The porosity and pore size distribution were analyzed by using mercury porosimetry (AutoPore IV 9500, Micromeritics, Shanghai, China). The bulk density was measured by the Archimedes method in water medium, as shown in Equation (1) [14]. Three independently measured density values are average out and reported as the density of the sintered sample:

(1) $$D_{b}=\frac{m_{1}D_{L}}{m_{3}-m_{2}}$$

where *D*_b_ refers to the bulk density, *D*_L_ represents the density of water, *m*_1_ corresponds to the mass of the sample dried in air, *m*_2_ denotes the mass of the sample in water and *m*_3_ refers to the mass of the sample with free bubbles on the surface.

## 3. Results and Discussion

**Figure 1:**
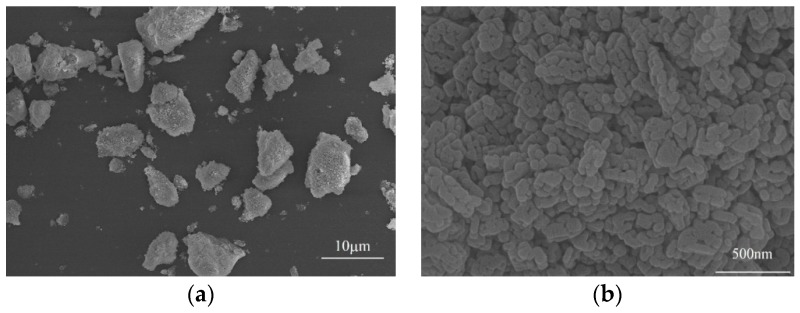
SEM micrographs of the high-activity magnesia powder (**a**) magnesia particles. and (**b**) surface of magnesia particle.

Figure 1 presents the microstructure of high-activity magnesia powder, which shows that the magnesia powder possesses smaller particle size and irregular shape. The magnesia particles have been formed due to the aggregation of a large number of plate-like magnesia microcrystals. Therefore, the magnesia particles possess a large number of internal pores and a high specific surface area.

**Figure 2:**
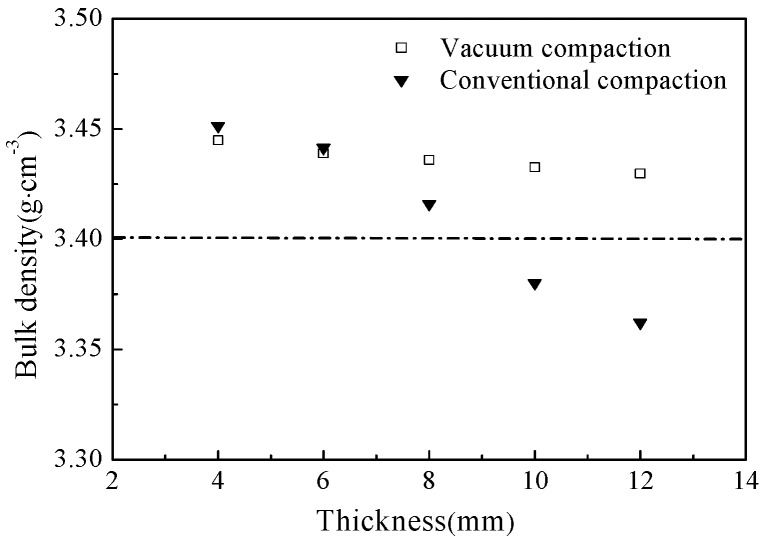
The bulk density of as-prepared samples, pressed by vacuum compaction and conventional compactions, with respect to thicknesses.

Figure 2 shows the changes in bulk density of the as-prepared samples, compacted by two different molding technologies and sintered at 1600 °C for 2 h, with respect to thickness. As shown in Figure 2, the bulk density of the samples, compressed by using conventional compaction method, significantly decreased with increasing thickness of magnesia green body and demonstrated a minimum thickness of 3.36 g·cm^−3^ at the thickness of 12 mm. Additionally, the bulk density had decreased below 3.40 g·cm^−3^, when the sample thickness exceeded 8 mm. However, the bulk density of the samples, molded by the vacuum compaction, exceeded 3.40 g·cm^−3^ even at the highest thickness of 12 mm. It is worth noting that both 8 mm thick samples have shown the bulk density of higher 3.40 g·cm^−3^, but the density of the vacuum-compacted sample was how much 0.6 percent higher than the conventionally-compacted sample. Thus, it can be concluded that entrapped air plays a critical role in determining the bulk density of magnesia and the bulk density decreases with increasing thickness of green body due to the entrapped air. Hence, the bulk density of magnesia can be effectively increased by using vacuum compaction technology, which removes the entrapped air from the green body.

**Figure 3:**
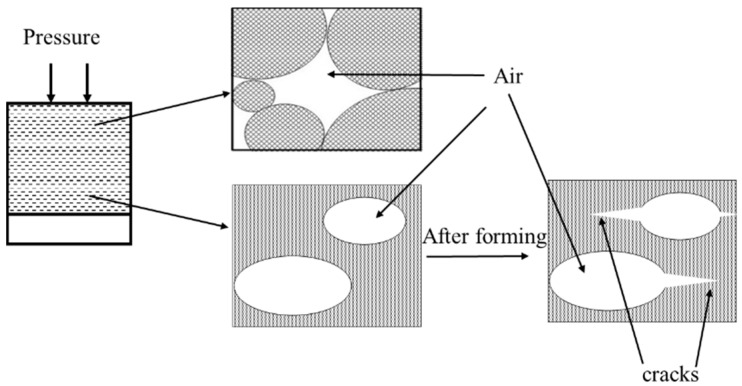
Schematic illustration of the entrapped-air induced defects inside the green body.

In general, the ceramic powders flow and rearrange under molding pressure [15]. However, under high molding pressure, a portion of the entrapped air cannot escape from the die and form internal pores. Based on the above results, an entrapped-air model for the green body can be schematically illustrated, as shown in Figure 3. It can be readily observed that the entrapped air forms internal pores in the green body. Moreover, the small particles cannot fill the gap between large particles due to the hindrance induced by entrapped air. In addition, the tensile stress of the internal air pressure produces an extension microcrack between the layers after the removal of molding pressure. The internal air pressure can be calculated by using Equation (2) [16]:

(2) $$P=P_{A}\frac{\rho_{P}\left(\rho_{\mathrm{th}}{-\rho }_{B}\right)}{\rho_{B}\left(\rho_{\mathrm{th}}{-\rho }_{P}\right)}$$

where *P* refers to the internal air pressure in the pore, *P*_A_ corresponds to the atmospheric pressure, ρ_th_ represents the theoretical density, ρ_P_ refers to the actual density of the green body and ρ_B_ denotes the packed density of raw materials. As shown in Equation (2), the bulk density and internal air pressure of green body increase with increasing applied pressure. However, during the vacuum compaction process, the original solid-air balance was broken in the die and internal air pressure was almost eliminated. Moreover, the smaller particles flow and fill the space between larger pores after air removal. Hence, the pore size of the vacuum-compacted green body become smaller and the contact area between particles become larger than the conventionally-pressed green body.

Furthermore, the uniformity of pores and contact area of particles play an important role in high-temperature densification of magnesia. The powder particles flow and form a contact area under high pressures. With increasing temperature, the necking of particles occurs and expands continuously in the contact region of powder particles due to the influence of surface tension [17]. At this time, the capillary force of the contact region between the particles dominates the sintering process, which can be calculated by Equation (3):

(3) $$\sigma =\gamma \left(\frac{1}{r_{n}}-\frac{1}{r}\right)$$

where σ represents the surface tension, γ denotes the surface free energy, *r*_n_ refers to the radius of the contact surface of the neck and *r* represents the radius of the curvature of the external surface of the necks. Equation (3) clearly demonstrates that the surface tension mainly depends on the radii of curvature of neck and contact surfaces. Moreover, the particles necking is facilitated by the large contact area between the powder particles.

In the later stages of sintering, the particles coalesced due to the grain growth and the stomatal coordination number decreased, both of which resulted in an increased bulk density of the magnesia samples [17]. Moreover, the magnesia particles started to bond together due to necking formation and expansion. As a result, the processes of the grain boundary migration and grain growth occurred at the same time. With increasing sintering time, the grain boundary migration significantly influenced the pores’ distribution under the action of interfacial energy. The grain boundaries crossed and wrapped the pores (smaller pores) when interfacial energy became higher than the threshold limit. One should note that the large diameter pores hindered the movement of grain boundaries and, in some cases (such as the interfacial energy was not enough), large pores have been observed between two or three grain boundaries. On the other hand, the conventionally-compacted samples have exhibited several microcracks due to the presence of entrapped air. Herein, the interfacial energy of grain boundary migration was not enough to expel these microcracks and various defects have been observed at grain boundaries. In summary, the vacuum compaction restrained the defect formation and increased the bulk density of as-prepared magnesia samples.

**Figure 4:**
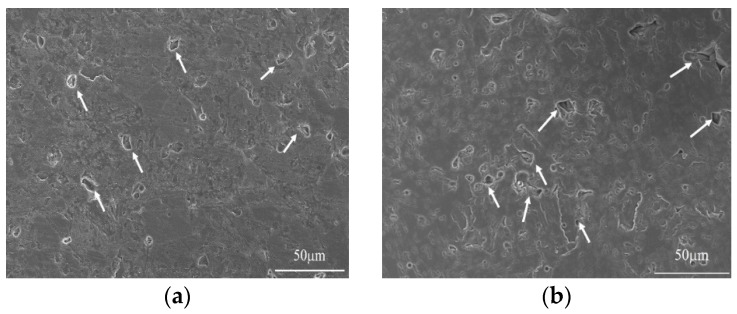

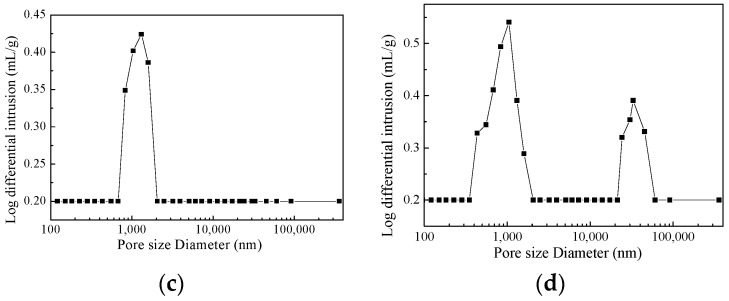
SEM micrographs and pore size distributions of samples with 8 mm using vacuum compaction molding (**a**,**c**) and conventional compaction molding (**b**,**d**).

Figure 4 presents the SEM images and pore size distributions of 8 mm thick samples, molded by using vacuum compaction and conventional compaction. It can be readily observed that the porosity of the vacuum-compacted samples was smaller with a uniform pore size distribution. In the vacuum-compacted sample, the pore size ranged from 830–1590 nm and most of the pores were either circular inner pores or smaller intergranular micropores. In conventionally-compacted magnesia sample, a small number of circular inner pores and large pores have been observed from the pore size distribution. Moreover, the pore size distribution has exhibited two distinct ranges; 434–1590 nm and 24,200–45,400 nm, which further confirmed that the entrapped air resulted in larger pores and decreased the bulk density of as-prepared magnesia sample (Figure 2). It is worth mentioning that the presence of large-sized pores damages the quality of magnesia and reduces its service life.

**Figure 5:**
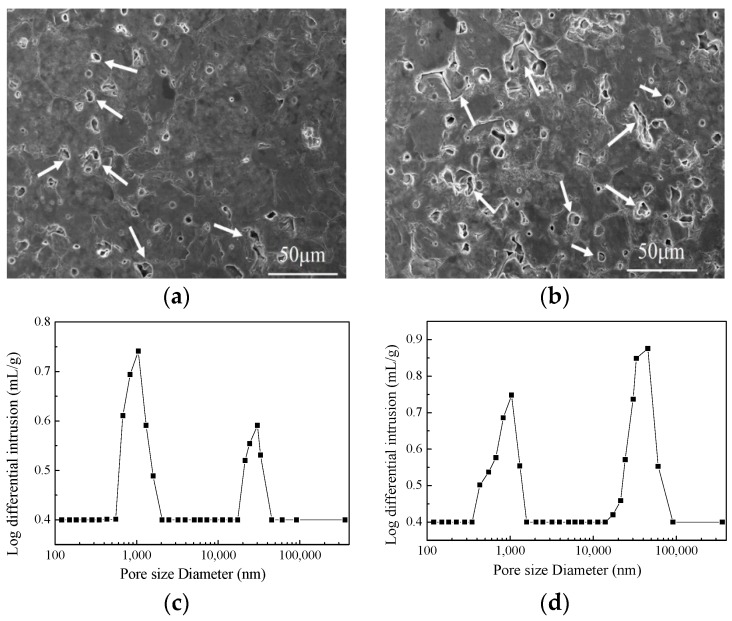
SEM micrographs and pore size distributions of samples with 12 mm using vacuum compaction molding (**a**,**c**) and conventional compaction molding (**b**,**d**).

Figure 5 shows the SEM images and pore size distributions of 12 mm thick samples, which are formed by using vacuum compaction and conventional compaction. It can be clearly observed from Figure 5a,b that the intergranular cracks are present at grain boundaries and the pore size exhibited two distinct ranges. The reason may be that the air between the powder particles was not completely removed or the applied pressure was not uniformly distributed between powders. Nevertheless, compared with the vacuum-compacted samples, the pore size of the conventionally-compacted sample was larger and a number of intergranular cracks have been observed. These observations indicate that the defects concentration increases with increasing molding thickness and results in an inferior bulk density of the samples. However, the vacuum compaction can deliver the magnesia samples with desirable density (3.40 g∙cm^−3^) at higher thicknesses of the green bodies. Hence, the utilization of proposed vacuum compaction has the potential to increase the production efficiency of sintered magnesia samples.

## 4. Conclusions

In summary, two different compaction methodologies, i.e., vacuum compaction and conventional compaction, have been adopted to obtained dense magnesia samples with different thicknesses. The influence of compaction method on bulk density and pore formation mechanism has been systematically explored. The main conclusions of the current study are as follow:

- The entrapped-air induced defects in magnesia samples resulted in large pores and intergranular microcracks, which decreased the bulk density of as-sintered magnesia samples. Moreover, the bulk density decreased with increasing thickness of the green body and a minimum bulk density of 3.36 g·cm^−3^ has been exhibited by 12 mm thick conventionally-compacted magnesia sample.
- The vacuum compaction effectively minimized the entrapped-air induced defects and resulted in a higher bulk density of the as-sintered magnesia samples. One should note that the bulk density of vacuum-compacted magnesia remained higher than 3.4 g·cm^−3^ for all thicknesses and the samples contained small circular pores.
- Even though the conventional compaction of 8 mm thick magnesia sample resulted in a higher bulk density of 3.40 g·cm^−3^, the presence of large pores and intergranular cracks is detrimental to the service life of magnesia.

## Figures and Tables

**Table 1:** Chemical composition of magnesite (wt %).

MgO	SiO_2_	Al_2_O_3_	Fe_2_O_3_	CaO	I. L.
47.03	0.26	0.06	0.27	0.66	51.72
